# WSES guidelines for management of *Clostridium difficile* infection in surgical patients

**DOI:** 10.1186/s13017-015-0033-6

**Published:** 2015-08-20

**Authors:** Massimo Sartelli, Mark A. Malangoni, Fikri M. Abu-Zidan, Ewen A. Griffiths, Stefano Di Bella, Lynne V. McFarland, Ian Eltringham, Vishal G. Shelat, George C. Velmahos, Ciarán P. Kelly, Sahil Khanna, Zaid M. Abdelsattar, Layan Alrahmani, Luca Ansaloni, Goran Augustin, Miklosh Bala, Frédéric Barbut, Offir Ben-Ishay, Aneel Bhangu, Walter L. Biffl, Stephen M. Brecher, Adrián Camacho-Ortiz, Miguel A. Caínzos, Laura A. Canterbury, Fausto Catena, Shirley Chan, Jill R. Cherry-Bukowiec, Jesse Clanton, Federico Coccolini, Maria Elena Cocuz, Raul Coimbra, Charles H. Cook, Yunfeng Cui, Jacek Czepiel, Koray Das, Zaza Demetrashvili, Isidoro Di Carlo, Salomone Di Saverio, Irina Magdalena Dumitru, Catherine Eckert, Christian Eckmann, Edward H. Eiland, Mushira Abdulaziz Enani, Mario Faro, Paula Ferrada, Joseph Derek Forrester, Gustavo P. Fraga, Jean Louis Frossard, Rita Galeiras, Wagih Ghnnam, Carlos Augusto Gomes, Venkata Gorrepati, Mohamed Hassan Ahmed, Torsten Herzog, Felicia Humphrey, Jae Il Kim, Arda Isik, Rao Ivatury, Yeong Yeh Lee, Paul Juang, Luis Furuya-Kanamori, Aleksandar Karamarkovic, Peter K Kim, Yoram Kluger, Wen Chien Ko, Francis D. LaBarbera, Jae Gil Lee, Ari Leppaniemi, Varut Lohsiriwat, Sanjay Marwah, John E. Mazuski, Gokhan Metan, Ernest E. Moore, Frederick Alan Moore, Carl Erik Nord, Carlos A. Ordoñez, Gerson Alves Pereira Júnior, Nicola Petrosillo, Francisco Portela, Basant K. Puri, Arnab Ray, Mansoor Raza, Miran Rems, Boris E. Sakakushev, Gabriele Sganga, Patrizia Spigaglia, David B. Stewart, Pierre Tattevin, Jean Francois Timsit, Kathleen B. To, Cristian Tranà, Waldemar Uhl, Libor Urbánek, Harry van Goor, Angela Vassallo, Jean Ralph Zahar, Emanuele Caproli, Pierluigi Viale

**Affiliations:** Department of Surgery, Macerata Hospital, Via Santa Lucia 2, 62019 Macerata, Italy; American Board of Surgery, Philadelphia, USA; Department of Surgery, College of Medicine and Health Sciences, UAE University, Al-Ain, United Arab Emirates; Department of Surgery, Queen Elizabeth Hospital, Birmingham, UK; 2nd Infectious Diseases Division, National Institute for Infectious Diseases L. Spallanzani, Rome, Italy; Department of Medicinal Chemistry, School of Pharmacy, University of Washington, Washington, USA; Department of Medical Microbiology, King’s College Hospital, London, UK; Department of Surgery, Tan Tock Seng Hospital, Singapore, Singapore; Emergency Surgery, and Surgical Critical Care, Massachusetts General Hospital, Harvard Medical School, Boston, MA USA; Gastroenterology Division, Beth Israel Deaconess Medical Center, Harvard Medical School, Boston, MA USA; Division of Gastroenterology and Hepatology, Department of Medicine, Mayo Clinic, Rochester, MN USA; Department of Surgery, University of Michigan, Ann Arbor, MI USA; Department of Obstetrics and Gynecology, Wayne State University, Detroit, MI USA; General Surgery I, Papa Giovanni XXIII Hospital, Bergamo, Italy; Department of Surgery, University Hospital Center Zagreb and School of Medicine, University of Zagreb, Zagreb, Croatia; Trauma and Acute Care Surgery Unit, Hadassah Hebrew University Medical Center, Jerusalem, Israel; UHLIN (Unité d’Hygiène et de Lutte contre les Infections Nosocomiales) National Reference Laboratory for Clostridium difficile Groupe Hospitalier de l’Est Parisien (HUEP), Paris, France; Department of General Surgery, Rambam Health Care Campus, Haifa, Israel; Academic Department of Surgery, Queen Elizabeth Hospital, Edgbaston, Birmingham, UK; Department of Surgery, University of Colorado, Denver Health Medical Center, Denver, USA; Pathology and Laboratory Medicine, VA Boston Healthcare System, West Roxbury MA and BU School of Medicine, Boston, MA USA; Department of Internal Medicine, University Hospital, Dr.José E. González, Monterrey, Mexico; Department of Surgery, University of Santiago de Compostela, Santiago de Compostela, Spain; Department of Pathology, University of Alberta Edmonton, Edmonton, AB Canada; Emergency Surgery Department, Maggiore Parma Hospital, Parma, Italy; Department of General Surgery, Medway Maritime Hospital, Gillingham Kent, UK; Department of Surgery, Division of Acute Care Surgery, University of Michigan, Ann Arbor, MI USA; Department of Surgery, Northeast Ohio Medical University, Summa Akron City Hospital, Akron, OH USA; Faculty of Medicine, Transilvania University, Infectious Diseases Hospital, Brasov, Romania; Division of Trauma, Surgical Critical Care, Burns, and Acute Care Surgery, University of California San Diego Health Science, San Diego, USA; Division of Acute Care Surgery, Trauma and Surgical Critical Care, Department of Surgery, Beth Israel Deaconess Medical Center, Harvard Medical School, Boston, MA USA; Department of Surgery,Tianjin Nankai Hospital, Nankai Clinical School of Medicine, Tianjin Medical University, Tianjin, China; Department of Infectious Diseases, Jagiellonian University, Medical College, Kraków, Poland; Department of General Surgery, Adana Numune Training and Research Hospital, Adana, Turkey; Department of Surgery, Tbilisi State Medical University, Kipshidze Central University Hospital, Tbilisi, Georgia; Department of Surgery, Hamad General Hospital, Doha, Qatar; Trauma Surgery Unit, Maggiore Hospital, Bologna, Italy; Clinical Infectious Diseases Hospital, Ovidius University, Constanta, Romania; National Reference Laboratory for Clostridium difficile, AP-HP, Saint-Antoine Hospital, Paris, France; Department of General, Visceral and Thoracic Surgery, Klinikum Peine, Hospital of Medical University Hannover, Peine, Germany; Vital Care, Inc, Meridian, MS USA; Department of Medicine, Section of Infectious Diseases, King Fahad Medical City, Riyadh, Saudi Arabia; Department of General Surgery, Trauma and Emergency Surgery Division, ABC Medical School, Santo André, SP Brazil; Division of Trauma, Critical Care and Emergency Surgery, Virginia Commonwealth University, Richmond, VA USA; Department of Surgery, Stanford University, Stanford, CA USA; Division of Trauma Surgery, Hospital de Clinicas, School of Medical Sciences, University of Campinas, Campinas, Brazil; Service of Gastroenterology and Hepatology, Geneva University Hospital, Genève, Switzerland; Critical Care Unit, Instituto de Investigación Biomédica de A Coruña (INIBIC), Complexo Hospitalario Universitario de A Coruña (CHUAC), Sergas, Universidade da Coruña (UDC), A Coruña, Spain; Department of Surgery Mansoura, Faculty of Medicine, Mansoura University, Mansoura, Egypt; Surgery Department, Hospital Universitario (HU) Terezinha de Jesus da Faculdade de Ciencias Medicas e da Saude de Juiz de Fora (SUPREMA), Hospital Universitario (HU) Universidade Federal de Juiz de Fora (UFJF), Juiz de Fora, Brazil; Department of Internal Medicine, Pinnacle Health Hospital, Harrisburg, PA USA; Department of Medicine, Milton Keynes University Hospital NHS Foundation Trust, Milton Keynes, Buckinghamshire UK; Department of Surgery, St. Josef Hospital, Ruhr University Bochum, Bochum, Germany; Department of Gastroenterology and Hepatology, Ochsner Clinic Foundation, New Orleans, LA USA; Department of Surgery, Ilsan Paik Hospital, Inje University College of Medicine, Goyang, Republic of Korea; General Surgery Department, Erzincan University Mengücek Gazi Training and Research Hospital, Erzincan, Turkey; School of Medical Sciences, Universiti Sains Malaysia, Kota Bharu, Kelantan Malaysia; Department of Pharmacy Practice, St Louis College of Pharmacy, St Louis, MO USA; Research School of Population Health, The Australian National University, Acton, ACT Australia; Clinic For Emergency surgery, University Clinical Center of Serbia, Faculty of Medicine University of Belgrade, Belgrade, Serbia; General and Trauma Surgery, Albert Einstein College of Medicine, North Bronx Healthcare Network, Bronx, NY USA; Division of Infectious Diseases, Department of Internal Medicine, National Cheng Kung University Hospital, Tainan, Taiwan; Division of Critical Care & Trauma Surgery, Department of Surgery, Yonsei University College of Medicine, Seoul, South Korea; Abdominal Center, Helsinki University Hospital Meilahti, Helsinki, Finland; Department of Surgery, Faculty of Medicine Siriraj Hospital, Mahidol University, Bangkok, Thailand; Department of Surgery, Post-Graduate Institute of Medical Sciences, Rohtak, India; Department of Surgery, Washington University School of Medicine, Saint Louis, USA; Department of Infectious Diseases and Clinical Microbiology, Hacettepe University Faculty of Medicine, Ankara, Turkey; Department of Surgery, University of Florida, Gainesville, FL USA; Department of Laboratory Medicine, Karolinska Institute, Karolinska University Hospital, Stockholm, Sweden; Department of Surgery, Fundación Valle del Lili, Hospital Universitario del Valle, Universidad del Valle, Cali, Colombia; Emergency Surgery and Trauma Unit, Department of Surgery, Ribeirão Preto, Brazil; Gastroenterology Department, Centro Hospitalar e Universitário de Coimbra, Coimbra, Portugal; Department of Medicine, Hammersmith Hospital and Imperial College London, London, UK; Infectious Diseases and Microbiology Unit, Milton Keynes University Hospital NHS Foundation Trust, Milton Keynes, Buckinghamshire UK; Department of Abdominal and General Surgery, General Hospital Jesenice, Jesenice, Slovenia; Department of Surgery, Medical University of Plovdiv, Plovdiv, Bulgaria; Division of General Surgery and Organ Transplantation, Department of Surgery, Catholic University of the Sacred Heart, Rome, Italy; Department of Infectious, Parasitic and Immune-Mediated Diseases, Istituto Superiore di Sanità, Rome, Italy; Department of Surgery, The Pennsylvania State University, College of Medicine, Hershey, PA USA; Infectious Diseases and Intensive Care Unit, Pontchaillou University Hospital, Rennes, France; AP-HP Bichat hospital, Medical and infectious diseases ICU, Paris, France; Emergency Medicine and Surgery, Macerata hospital, Macerata, Italy; 1st Surgical Clinic, University Hospital of St. Ann Brno, Brno, Czech Republic; Department of Surgery, Radboud University Medical Center, Nijmegen, Netherlands; Infection Prevention/Epidemiology, Providence Saint John’s Health Center, Santa Monica, CA USA; Infection Control Unit, Angers University, CHU d’Angers, Angers, France; Department of Surgery, Ancona University Hospital, Ancona, Italy; Clinic of Infectious Diseases, St Orsola-Malpighi University Hospital, Bologna, Italy

## Abstract

In the last two decades there have been dramatic changes in the epidemiology of *Clostridium difficile* infection (CDI), with increases in incidence and severity of disease in many countries worldwide. The incidence of CDI has also increased in surgical patients. Optimization of management of *C difficile*, has therefore become increasingly urgent. An international multidisciplinary panel of experts prepared evidenced-based World Society of Emergency Surgery (WSES) guidelines for management of CDI in surgical patients.

## Executive summary

In the last two decades, the dramatic increase in incidence and severity of *Clostridium difficile* infection (CDI) in many countries worldwide [1], has made CDI a global public health challenge [2–5]. Recently two comprehensive sets of guidelines for management of CDI were published [6, 7] that do not address issues specifically with regard to surgeons. CDI in surgical patients is of particular interest. Surgery, especially gastrointestinal surgery, may predispose patients to the development of CDI. Surgery is also a treatment option in severe cases of CDI [8–11]. Optimization of the perioperative CDI patient management is therefore necessary for reduction in health care costs, as well as patient morbidity and mortality. To provide empirical guidelines for the surgeon called upon to assist in the care of the CDI patient, an international multidisciplinary panel of experts worldwide have prepared these evidenced-based guidelines for the management of *C. difficile* infection. In constituting the expert panel, the board of World Society of Emergency Surgery (WSES) involves many of the world’s leading surgical experts in management of CDI. This expert panel includes professionals who treat CDI patients on a daily basis as well as those with research interests in the condition. These guidelines outline clinical recommendations based on the Grading of Recommendations Assessment, Development, and Evaluation (GRADE) hierarchy criteria summarized in Table 1 [12, 13].

**Table 1:** Grading of recommendations from Guyatt and colleagues [12, 13]

Grade of recommendation	Clarity of risk/benefit	Quality of supporting evidence	Implications
1A			
Strong recommendation, high-quality evidence	Benefits clearly outweigh risk and burdens, or vice versa	RCTs without important limitations or overwhelming evidence from observational studies	Strong recommendation, applies to most patients in most circumstances without reservation
1B			
Strong recommendation, moderate-quality evidence	Benefits clearly outweigh risk and burdens, or vice versa	RCTs with important limitations (inconsistent results, methodological flaws, indirect analyses or imprecise conclusions) or exceptionally strong evidence from observational studies	Strong recommendation, applies to most patients in most circumstances without reservation
1C			
Strong recommendation, low-quality or very low-quality evidence	Benefits clearly outweigh risk and burdens, or vice versa	Observational studies or case series	Strong recommendation but subject to change when higher quality evidence becomes available
2A			
Weak recommendation, high-quality evidence	Benefits closely balanced with risks and burden	RCTs without important limitations or overwhelming evidence from observational studies	Weak recommendation, best action may differ depending on the patient, treatment circumstances, or social values
2B			
Weak recommendation, moderate-quality evidence	Benefits closely balanced with risks and burden	RCTs with important limitations (inconsistent results, methodological flaws, indirect or imprecise) or exceptionally strong evidence from observational studies	Weak recommendation, best action may differ depending on the patient, treatment circumstances, or social values
2C			
Weak recommendation, Low-quality or very low-quality evidence	Uncertainty in the estimates of benefits, risks, and burden; benefits, risk, and burden may be closely balanced	Observational studies or case series	Very weak recommendation; alternative treatments may be equally reasonable and merit consideration

### Recommendations

#### Diagnosis

1) Stool testing should only be performed on diarrhea stools from at-risk patients with clinically significant diarrhea (Recommendation 1 C).

2) For patients with ileus who may be unable to produce stool specimens, polymerase chain reaction testing of perirectal swabs may be an accurate and efficient method to detect toxigenic *C. difficile* in patients with symptoms of CDI (Recommendation 2B).

3) Nucleic acid amplification tests (NAAT) such as polymerase chain reaction (PCR) for *C. difficile* toxin genes appear to be sensitive and specific and may be used as a standard diagnostic test for CDI. NAAT as single-step algorithm can increase detection of asymptomatic colonization therefore it should only be performed in patients with clinical suspicion for CDI (Recommendation 1 B).

4) Glutamate dehydrogenase (GDH) screening tests for *C. difficile* are sensitive but do not differentiate between toxigenic and non-toxigenic strains. They may be used in association with toxin A and B EIA testing. Algorithms involving screening with an EIA for GDH followed by a toxin assay may be used (Recommendation 1 B).

5) Enzyme immunoassay (EIA) for toxin A/B is fast and inexpensive and has high specificity but it is not recommended alone due to its relatively low sensitivity. (Recommendation 1 B).

6) *Clostridium difficile* culture is relatively slow but sensitive. It is rarely performed today as a routine diagnostic test. *C. difficile* culture is recommended for subsequent epidemiological typing and characterization of strains (Recommendation 1 C).

7) Repeat testing within 7 days should not be performed on patients who previously tested negative unless the clinical picture has changed significantly (Recommendation 1 C).

8) Immunocompromised patients (including patients in chemotherapy, chronic corticosteroid therapy or immunosuppressive agents, and post-transplant patients) should be always tested for CDI if they have a diarrheal illness (Recommendation 1 C).

9) CT imaging is suggested for suspected severe-complicated *C. difficile* colitis, however its sensitivity is not satisfactory for screening purposes (Recommendation 2 B).

10) Ultrasound may be useful in critically ill patients suspected to have pseudomembranous colitis who cannot be transported for CT scan (Recommendation 2 C).

11) Flexible sigmoidoscopy may be helpful for the diagnosis of *C. difficile* colitis (CDC) when there is a high level of clinical suspicion for *C. difficile* despite repeated negative laboratory assays (Recommendation 2 B).

#### *Antimicrobial therapy*

12) Unnecessary antimicrobial agent(s) and proton pump inhibitors should be discontinued if CDI is suspected (Recommendation 1 C).

13) Empirical therapy for CDI should be avoided unless there is a strong suspicion for CDI. If a patient has a strong suspicion for CDI, empirical therapy for CDI should be considered while awaiting test results (Recommendation 1 B).

14) Metronidazole is recommended for the treatment of mild-moderate disease (Recommendation 1 A).

15) Oral vancomycin is recommended for treatment of patients with severe disease, or for patients with mild-moderate disease who do not respond to metronidazole. (Recommendation 1 A).

16) In patients in whom oral antibiotics cannot reach the colon, vancomycin may be administered by enema and metronidazole can be given intravenously (Recommendation 1 B).

17) Fidaxomicin may be used to treat CDI, especially in the patients at higher risk for recurrence (e.g. elderly patients with severe underlying disease or those requiring receiving concomitant antibiotics) (Recommendation 1 A).

#### Surgical management

18) Patients with severe CDI who progress to systemic toxicity should undergo early surgical consultation and evaluated for potential surgical intervention (Recommendation 1 C).

19) Resection of the entire colon should be considered to treat patients with fulminant colitis (FC) (Recommendation 1 B).

20) Diverting loop ileostomy with colonic lavage may be a useful alternative to resection of entire colon (Recommendation 2 C).

21) Patients with FC should be treated with high dose oral or by enema vancomycin (500 mg, 6 hourly) in combination with intravenous metronidazole (500 mg, 8 hourly). (Recommendation 1 C).

#### Supportive care

22) Supportive measures, including intravenous fluid resuscitation and electrolyte replacement, should be provided to all patients with severe *C. difficile* infection (Recommendation 1 C).

23) Early detection of shock and aggressive management of underlying organ dysfunction are essential for optimum outcomes in patients with fulminant colitis (Recommendation 1 C).

#### Recurrent C. difficile infection (RCDI)

24) Agents that may be used to treat the first recurrence of CDI include metronidazole, for non-severe RCDI, and vancomycin for severe RCDI. (Recommendation 1 B).

25) Fidaxomicin may be used as an alternative agent (Recommendation 1 B).

26) In subsequent recurrence of CDI (2nd or later) oral vancomycin or fidaxomicin is recommended (Recommendation 1 B).

#### Probiotics

27) Probiotics may be considered as an adjunctive treatment to antibiotics for immunocompetent patients with RCDI (Recommendation 2 B).

#### Faecal microbiota transplantation

28) Intestinal or faecal microbiota transplantation (IMT or FMT) may be an effective option for the treatment of RCDI (Recommendation 1 B).

29) FMT may be effective in immunocompromised patients and patients who have had solid organ transplants (Recommendation 2 B).

#### Intravenous immunoglobulin (IVIG)

30) IVIG should only be used as adjunct therapy in patients with multiple recurrent or fulminant CDI until results from large, randomized controlled trials are available (Recurrence 2 C).

#### Monoclonal antibodies

31) Infusion with monoclonal antibodies may be of use to prevent recurrences of CDI, particularly in patients with CDI due to the 027 epidemic strain (Recommendation 2 C).

#### Enteral nutrition in CDI

32) Tube feeding patients should be clinically assessed due to their risk for developing CDI (Recommendation 2 C).

#### Anti-motility agents

33) The use of anti-peristaltic agents for the treatment of CDI should be discouraged. If anti-peristaltic, if used in isolation agents, are used to control persistent symptoms in patients with CDI they must always be accompanied by medical therapy (Recommendation 2 C).

#### Prevention

34) Proper antimicrobial stewardship in selecting an appropriate antibiotic and optimizing its dose and duration to cure an infection may prevent the emergence of *C. difficile* (Recommendation 1 B).

35) Patients with suspected or proven CDI should be placed in contact (enteric) precautions (Recommendation 1 B).

36) Hand hygiene with soap and water is a cornerstone of the prevention of *C. difficile*. Hand hygiene, contact precautions and good cleaning and disinfection of the environment and patient care equipment, should be used by all health-care workers contacting any patient with known or suspected CDI (Recommendation 1 B).

## Introduction

*C. difficile* is an anaerobic, spore forming Gram-positive bacillus, which may form part of the normal intestinal microbiota in healthy newborns but which is rarely present in the gut of healthy adults [14–16]. The organism is spread via the oral-fecal route and in hospitalized patients may be acquired through the ingestion of spores or vegetative bacteria spread to patients by healthcare personnel’s hands or from the environment [17, 18]. It is the most common cause of diarrhea in hospitalized patients.

## Pathogenesis

*Clostridium difficile* spores survive the acidic environment of the stomach and germinate in the intestine [19]. They act as an environmental reservoir for *C. difficile* and can facilitate spread among patients, as well as contributing to the high recurrence rates observed in CDI. The primary toxins produced by this bacterium are toxins A and B [20]. Some strains of *C. difficile* also produce binary toxin. Toxins A and B act as glucosyltransferases, promoting the activation of Rho GTPases leading to disorganization of the cytoskeleton of the colonocyte, and eventual cell death [21]. Since CDI is a toxin mediated infection, non toxigenic *C. difficile* strains are non-pathogenic. Over the years the respective roles and importance toxins A and B has been debated. Toxin A was thought to be the major virulence factor for many years, [22–24]. It is now established that both toxins A and B are important for inducing colonocyte death and colitis. In addition to toxins A and B, some strains produce a third toxin known as binary toxin [25–29]. Binary toxin has an ADP-ribosyltransferase function, which also leads to actin depolymerization [30, 31]. It has been demonstrated in *C. difficile* strains associated with nosocomial outbreaks of CDI with increased clinical severity [32, 33].

Typing is useful to differentiate *C. difficile* strains and to obtain epidemiological information. Different typing methods for *C. difficile* are actually available: restriction endonuclease analysis (REA), pulsed-field gel electrophoresis (PFGE), multilocus sequence typing (MLST), repetitive-element PCR typing, toxinotyping, multilocus variable-number tandem-repeat analysis (MLVA) and PCR- ribotyping [34].

*C. difficile* strains with increased virulence traits (hyper virulent), have been described in the last 10 years. In particular, PCR-ribotype 027, also known as North American pulsed-field gel electrophoresis type 1 (NAP1) or restriction endonuclease analysis group BI, has been associated with increased disease severity, recurrence and significant mortality [35].

Asymptomatic colonization may occur in 6 to 50 % of long-term care facility residents depending on whether CDI is endemic [36, 37]. In a 15-month prospective study of 4143 patients performed in six Canadian hospitals in Quebec and Ontario [38], 184 (4.4 %) had asymptomatic colonization at the time of unit admission, and 123 (3.0 %) had health care–associated *C. difficile* colonization.

## Risk factors

Risk factors for CDI may be divided into three general categories: host factors (immune status, co-morbidities), exposure to CD spores (hospitalizations, community sources, long-term care facilities) and factors that disrupt normal colonic microbiome (antibiotics, other medications, surgery) [39].

## Host factors

Risk factors identified to date include, age more than 65 years, comorbidity or underlying conditions, inflammatory bowel diseases, immunodeficiency (including human immunodeficiency virus infection, hematologic malignancies and chemotherapy), malnutrition, and low serum albumin level [3, 40]. Diabetes mellitus is increasingly recognized as a risk factor for hospital and community-acquired CDI [41]. More recently, gene polymorphisms (e.g. IL-8) may be associated with increased risk for CDI but further studies are needed [42].

The effect of prior appendectomy on the development of *C. difficile* colitis has been debated in literature [43].

A recent review by Seretis et al. [44] of five studies conducted retrospectively was published in 2014. Although the results were conflicting regarding the impact of prior appendectomy on the occurrence or relapse of CDI, it appeared that an *in situ* appendix did not impact on the development of CDI.

In the retrospective analysis by Clanton et al. [45] on 55 patients who underwent colectomy for CDI between 2001 and 2011, a prior appendectomy was noted in 24 of 55 specimens (44, 99 % CI: 0.280–0.606). This was compared to an observed lifetime rate of appendectomy of 17.6 %. The rate of appendectomy in the cohort of patients who later underwent colectomy for CDI was significantly higher than would be expected in the general population (44 % vs 18 %, P < 0.01).

In a second retrospective study [46], of 388 patients with an intact appendix, 20 (5.2 %) developed fulminant infection and required colectomy, whereas of the 119 patients with a previous appendectomy, 13 (10.9 %) required colectomy. An increased severity of disease, indicated by increased rate of colectomy, occurred for the group with a history of appendectomy (*P* = 0.03).

A sub-group analysis of a large population based study published in 2013 [47] showed that appendectomy was not associated with adverse outcomes in CDI. Patients with appendectomy before CDI had no differences in risk factors, treatment, or outcomes including treatment failure, development of severe or severe-complicated CDI, and recurrence rates as compared with patients without appendectomy.

Larger prospective studies are needed to assess the impact of prior appendectomy on development and severity of CDI.

## Exposure to *Clostridium difficile* spores

Factors that increase risk of exposure to *C. difficile* spores, such as increased duration of hospital stay may increase the risk of CDI. A length of stay > 2 weeks has been shown to be a risk factor for CDI [48]. Hospitals with well implemented infection prevention and control measures may reduce the risk of patients of developing CDI [49].

## Normal flora disruption

The indigenous gut microbiota is the complex community of microorganisms that populates the gastrointestinal tract. This micro-ecosystem plays a crucial role in protecting the intestines by providing resistance to colonization and infection by pathogenic organisms [50]. Gut microbiota also has immeasurable effects on homeostasis in the host [51]. Under normal conditions, the human gut microbiota may impede pathogen colonisation through general mechanisms such as direct inhibition through bacteriocins, nutrient depletion (consuming growth-limiting nutrients) or stimulation of host immune defences [38] though the exact mechanism by which the microbiota protects against CDI is unknown [52]. Disruption of the normal balance of colonic microbiota as a consequence of antibiotic use or other stressors, is, however, likely to be important [53].

### Antibiotic exposure

It is presumabed that disruption of the normal gut flora provides a perfect setting for *C. difficile* to proliferate and produce toxin.

The risk of CDI is increased up to 6-fold during and in the subsequent month after antibiotic therapy [54]. Although nearly all antibiotics have been associated with CDI, clindamycin, third-generation cephalosporins, penicillins and fluoroquinolones have traditionally been considered to pose the greatest risk [55–61]. An association between CDI and antimicrobial treatment > 10 days has also been demonstrated [62, 63]. Antibiotics which have been less commonly associated with CDI include macrolides, sulfonamides and tetracyclines [64]. Even very limited exposure, such as single-dose surgical antibiotic prophylaxis may increase patients risk for both *C. difficile* colonization [65, 66] or infection.

### Other medications

Exposure to gastric acid-suppressive medications, such as histamine-2 blockers and proton pump inhibitors (PPIs) may be a potential risk factor for development of CDI. Recent studies have suggested the association between use of stomach acid–suppressive medications, primarily PPIs, and CDI [67, 68]. In 2012 a systematic review of [69] 42 observational studies (30 case–control, 12 cohort) totalling 313,000 participants were evaluated for incident and recurrent CDI in PPI users. Despite the substantial statistical and clinical heterogeneity, the findings indicated a probable association between PPI use and incident and recurrent CDI. This risk was further increased by concomitant use of antibiotics and PPI. Other studies suggested that this association may be the result of confounding with the underlying severity of illness and duration of hospital stay [70]. Given that acid suppression drugs, especially PPIs, may be over- prescribed in surgical settings consideration should be given to stopping PPIs in patients at high risk of CDI.

### Surgery

Recent reports have linked the development of CDI in surgical patients with widespread use of broad-spectrum antibiotics, increasing numbers of elderly and immunocompromised patients undergoing surgical interventions and the emergence of more virulent strains of *C. difficile* [8, 71, 72].

Abdelsattar et al. [11] prospectively identified patients with laboratory-confirmed postoperative CDI after different general, vascular, or gynaecological surgeries at 52 academic and community hospitals in the state of Michigan, USA between July 2012 and September 2013. The highest rates of CDI occurred after lower-extremity amputation (2.6 %), followed by bowel resection or repair (0.9 %) and gastric or esophageal operations (0.7 %). Gynaecological and endocrine operations had the lowest rates (0.1 and 0 %, respectively). Using multivariable analyses, older age, chronic immunosuppression, hypoalbuminemia (≤3.5 g/dL) and preoperative sepsis were associated with CDI. Use of prophylactic antibiotics was not independently associated with CDI, neither was sex, body mass index (BMI), surgical priority, weight loss, or comorbid conditions.

Zerey et al. [8] performed a five-year retrospective analysis of the Agency for Healthcare Research and Quality’s National Inpatient Sample Database representing a stratified 20 % sample of hospitals in the United States, from 1999 to 2003. Patients undergoing an emergency operation were at higher risk of CDI than those having operations performed electively. Colectomy, small-bowel resection, and gastric resection were associated with the highest risk of CDI. Patients undergoing cholecystectomy and appendectomy had the lowest risk.

In 2010, Rodriguez et al. [73] published a retrospective analysis of all general surgery inpatients admitted to a large tertiary referral general surgical unit in the United Kingdom, between March 2005 and May 2007. Multivariate analysis identified malignancy, gastrointestinal disease, anemia, respiratory disease, circulatory disease, diabetes mellitus, those undergoing gastrointestinal surgery and increasing age to be independently associated with *C. difficile*.

To assess risk factors associated with CDI on a surgical ward, in 2012 Kim et al. conducted a retrospective chart review of all patients admitted between January 2010 and July 2011 [74]. The rate of CDI occurrence was 0.4 % (19/4,720 patients). Multivariate analysis showed that colectomy and hospital stays longer than 10 days were the highest risk factors for CDI occurrence in the surgical ward.

Using the Japanese Diagnosis Procedure Combination inpatient database, Yasunaga et al. [75] analyzed factors affecting the occurrence of CDI and the outcomes of CDI following digestive tract surgery. Of 143,652 patients undergoing digestive tract surgery, CDI was identified in 409 (0.28 %) patients. High mortality, long hospital stay and high costs were associated with postsurgical CDI.

Colo-rectal surgery is known as risk factor for CDI in surgical patients [76, 77]. Recently Damle et al. [78] published a retrospective analysis of patients who developed CDI following colorectal resection. Utilizing the U.S. University Health System Consortium database the authors identified adult patients undergoing colorectal surgery between 2008 and 2012. A total of 84,648 patients met study inclusion criteria. CDI occurred in 1,266 (1.5 %) patients during the study period. The strongest predictors of CDI were emergency procedure, inflammatory bowel disease, and severity of illness score. CDI was associated with a higher rate of complications, intensive care unit (ICU) admission, longer preoperative inpatient stay, 30-day readmission rate, and death within 30 days compared to non-CDI patients.

In 2008 Lumpkins et al. [79] published a retrospective observational study about the incidence of CDI in the critically injured trauma population. Five hundred eighty-one consecutive critically injured trauma patients were followed prospectively for development of CDI, diagnosed by toxin assay. Among 581 patients 19 cases of CDI were diagnosed (3.3 %). Intensive care unit length of stay, ventilator days, and hospital length of stay were significantly higher in the CDI patients. The diagnosis was made at mean of 17 days after admission; however, in four patients (21 %), the infections were diagnosed within six days of admission. Fourteen patients (74 %) had received therapeutic antibiotics for confirmed or suspected infection prior to the appearance of colitis; four patients (21 %) received only intraoperative prophylaxis, and one patient had no antibiotic exposure.

Recently Egorova et al. [80] reviewed the trend, hospital variability in CDI rates, in vascular surgery in USA. The rates of CDI after major vascular procedures including aortic abdominal aneurysm (AAA) repair, carotid endarterectomy or stenting, lower extremity revascularization (LER), and LE amputation were identified using Nationwide Inpatient Sample database for 2000–2011. During the study period the rates of CDI after vascular procedures had increased by 74 % from 0.6 in 2000 to 1.05 % in 2011. In 2011, the highest rates were after ruptured AAA repair (3.3 %), followed by lower extremity amputations (2.3 %), and elective open AAA (1.3 %).

### Iinflammatory bowel disease (IBD)

Patients with inflammatory bowel disease (IBD) may have increased risk of developing CDI, along with worse outcomes, higher rates of colectomy and higher rates of recurrence [81–84]. Patients with IBD also appear to have higher rates of asymptomatic carriage of *C. difficile* [85]. They receive various types of immunosuppressive drugs including steroids that has been found to increase the risk of CDI [86, 87].

The clinical presentation of an IBD exacerbation and CDI often is indistinguishable and requires a high index of suspicion for adequate treatment [6]. As the symptoms of CDI and an exacerbation of IBD (diarrhea, abdominal pain, fever and leukocytosis) overlap, the diagnosis of CDI may be delayed if it is not tested for [88]. In addition, in IBD patients with ileostomies, the development of acute enteritis as manifested by an increase in ileostomy output, nausea, fever and leukocytosis may also indicate CDI. The same is true for pouchitis, which presents as an increase in the number of stools per day [89]. In one study 10.7 % of patients with ileal pouch anal anastomosis, presenting with pouchitis, were found to have CDI [90].

In patients with IBD and severe colitis, empirical therapy directed against both CDI and treatment of an IBD flare should be started simultaneously while awaiting results of *C. difficile* testing [6].

Due to high rates of asymptomatic colonization of *C. difficile* in patients with IBD, only patients with increased diarrhea or new symptoms attributable to CDI should be tested for *C. difficile* toxin. Typical findings of CDI on colonoscopy are often absent in patients with IBD (0–13 % of cases) [91] which may be attributed to a weakened inflammatory response. There is no evidence from prospective studies to suggest that one antibiotic regimen is better than another for the treatment of CDI in IBD patients. Considering the worse outcomes seen in patients with IBD and CDI, some institutions use vancomycin as first line therapy. In a survey of North American gastroenterologists, there was no agreement on combination of antibiotics and immunomodulators in patients with an IBD flare and CDI [92]. The American College of Gastroenterology recommended with low quality supporting evidence, that ongoing immunosuppression can be maintained in patients with CDI but escalation of immunosuppression should be avoided.

Physicians should remain alert to the possibility of CDI in a patient with an IBD exacerbation to ensure rapid diagnosis and treatment. Early surgical consultation is also key for improving outcomes of patients with severe disease. Colectomy with preservation of the rectum may need to be considered for severely ill IBD patients with CDI.

### Immunocompromise patients

It is well known that the rate of CDI in the post-transplant setting is higher [93]. It has also been reported that cancer patients have a higher risk compared with non-cancer patients [94] due to chemotherapy causing the immunosuppression. Recently two retrospective studies were published on CDI in cancer patients [95, 96].

In the first a total of 225 patients were included, and 39 of them (17.3 %) were diagnosed with CDI. Type of tumor significantly differed between CDI patients, thus relative risk in each type of cancer was calculated after adjusting for age, antibiotic exposure, corticosteroid, and proton-pump inhibitor use. Patients with gastrointestinal tumors were less prone to CDI. Conversely, breast cancer patients have a greater predisposition to CDI. Antibiotic treatment was found to be associated with an increasing risk for CDI in breast cancer patients [95].

In the second study of 277 cancer patients with diarrhea 41 (14.8 %) were *C. difficile* toxin-positive. Multivariate analysis showed that chemotherapy (OR, 8.308; 95 % CI, 1.997–34.572; *P* = 0.004) and a positive result of fecal occult blood test (OR, 8.475; 95 % CI, 1.463–49.109; *P* = 0.017) were independent risk factors for acquisition of CDI among cancer patients [96].

Patients with HIV/AIDS are at a high risk of being infected with *C. difficile* too. This relationship is stronger in those with low absolute CD4 T cell counts or who meet clinical criteria for an AIDS diagnosis [97].

The increased risk may be partially attributed to frequent hospitalization, exposure to antibiotics and antibiotic prophylaxis for opportunistic infections, but HIV related alterations in fecal microbiota, gut mucosal integrity, and humoral and cell mediated immunity may be also likely to play a role [98].

## Community-acquired *C. difficile* infection (CA-CDI)

Community-acquired CDI (CA-CDI) has been demonstrated in populations previously thought to be at low-risk, including younger patients not previously exposed to antibiotics [99]. Suggested risk factors include increasing outpatient antibiotic prescriptions, greater use of acid-suppression medications, an increase in the proportion of asymptomatic carriers in the community and novel risk factors like food and water contamination [100]. A sub-group analysis of a population-based epidemiological study of CDI in Olmsted County, Minnesota from 1991–2005 was published in 2012 [101]. Of 157 CA-CDI cases, the median age was 50 years and 75.3 % were female. Among CA-CDI cases, 40 % required hospitalization, 20 % had severe and 4.4 % had severe-complicated infection, 20 % had treatment failure and 28 % had recurrent CDI.

Recently a systematic review and meta-analysis investigated the association between commonly prescribed medications and comorbidities with CA-CDI [41]. Twelve publications (*n* = 56,776 patients) met inclusion criteria. Antimicrobial (odds ratio, 6.18; 95 % CI 3.80–10.04) and corticosteroid (1.81; 1.15–2.84) exposure were associated with increased risk of CA-CDI. Among the comorbidities, inflammatory bowel disease (odds ratio, 3.72; 95 % CI, 1.52–9.12), renal failure (2.64; 1.23–5.68), hematologic cancer (1.75; 1.02–5.68), and diabetes mellitus (1.15; 1.05–1.27) were associated with CA-CDI. By location, antimicrobial exposure was associated with a higher risk of CA-CDI in the United States, whereas proton-pump inhibitor exposure was associated with a higher risk in Europe. By life stages, the risk of CA-CDI associated with antimicrobial exposure greatly increased in adults older than 65 years.

## Risk factors for recurrent CDI

In a meta-analysis by Garey et al. [102] found that continued use of non-*C. difficile* antibiotics after diagnosis of CDI (OR: 4.23; 95 % CI: 2.10–8.55; P < 0.001), concomitant receipt of antacid medications (OR: 2.15; 95 % CI: 1.13–4.08; *P* = 0.019), and older age (OR: 1.62; 95 % CI: 1.11–2.36; *P* = 0.0012) were significantly associated with an increased risk of recurrent CDI. Other factors identified in individual studies include age, hospital exposure, comorbid conditions, severe underlying illness, poor quality of life scores, initial disease severity and previous recurrent CDI [103, 104].

A recent systematic review and meta-analysis [105] was published to evaluate current evidence on the risk factors for recurrent CDI. A total of 33 studies (*n* = 18,530) met the inclusion criteria. The most frequent independent risk factors associated with recurrent CDI were age ≥65 years (risk ratio [RR], 1.63; 95 % confidence interval [CI], 1.24–2.14; *P* = .0005), additional antibiotics during follow-up (RR, 1.76; 95 % CI, 1.52–2.05; P < .001), use of proton-pump inhibitors (PPIs) (RR, 1.58; 95 % CI, 1.13–2.21; *P* = .008), and renal insufficiency (RR, 1.59; 95 % CI, 1.14–2.23; *P* = .007). The risk was also greater in patients previously on fluoroquinolones (RR, 1.42; 95 % CI, 1.28–1.57; P < .001).

## Clinical manifestations

The spectrum of symptomatic CDI ranges from mild diarrhea to severe disease or fulminant colitis and as many as 30 % of patients may develop recurrent CDI [106, 107].

Though diarrhea is the hallmark symptom of CDI it may not be present initially, possibly due to colonic dysmotility either from previous underlying conditions or possibly from the disease process itself [108].

This is especially important in surgical patients who may have a concomitant ileus. Therefore, in surgical patients it is important to have a high index of suspicion for the development of CDI.

## Mild-moderate CDI

Diarrhea may be accompanied by mild abdominal pain and cramps and if prolonged may result in altered electrolyte balance and dehydration. When this occurs in patients with severe comorbidity, particularly after surgery, non-severe CDI may increase morbidity significantly [109].

## Severe CDI

Severe CDI is associated with increased abdominal cramping and pain and constitutional features such as fever, leukocytosis, and hypoalbuminemia. The absence of diarrhoea in these patients may signal a progression to fulminant infection [110]. Though a wide variety of severity predictors for severe CDI has been described [111–115] international consensus for the definition of severe CDI is lacking [6, 7, 116].

One systematic review identifying risk factors for adverse outcomes of CDI was published by Abou Chakra et al. in 2012 [114]. Except for leukocytosis, albumin and age, there was much heterogeneity in the data and most studies were limited by small sample sizes.

To investigate the prognostic value of fever, leukocytosis, and renal failure, Bauer et al. [113] in 2012 analyzed the database of two randomized controlled trials, which contained information for 1105 patients with CDI. They found that both leucocytosis and renal failure were useful predictors of a complicated course of CDI. Miller et al. [115] in 2013 subsequently published an analysis of the same two clinical therapeutic trials to validate a categorization system to stratify CDI patients into severe or mild-moderate groups. A combination of five simple and commonly available clinical and laboratory variables (ATLAS) measured at the time of CDI diagnosis were able to accurately predict treatment response to CDI therapy. The ATLAS criteria included: age, treatment with systemic antibiotics, leucocyte count, albumin and serum creatinine [115].

Any of the following may be predictors of severe CDI:

- WBC >15 × 10^9^/L
- Acutely rising serum creatinine
- Temperature >38.5 °C
- Albumin <2.5 mg/dL

The progression to fulminant *C. difficile* colitis is relatively infrequent [109] (1–3 % of all CDI) although mortality in this group of patients remains high due to the development of toxic megacolon with colonic perforation, peritonitis and septic shock and subsequent organ dysfunction. Systemic symptoms may result from toxin-induced inflammatory mediators released locally in the colon [117–119]. Studies have demonstrated a significant rise in the number of cases of fulminant colitis associated with multiple organ failure and increased mortality in recent years associated with the hypervirulent 027 strain of *C. difficile* [120, 121]. Early diagnosis and treatment is therefore important in reducing the mortality associated with fulminant colitis. Patients who present with organ failure including increased serum lactate or vasopressor requirements, should be assessed immediately with regard to early operative intervention [121].

## Recurrent CDI (RCDI)

Recurrence of symptoms after initial therapy for *C. difficile*, develops in 10–30 % of cases, and this often presents a clinical challenge. Patients may have several episodes of recurrence that may occur over a period of years [122–127]. Recurrence and reinfection are therefore difficult to distinguish by symptoms alone, but may be distinguished if the strain of *C. difficile* is typed.

RCDI may be either a consequence of germinating resident spores remaining in the colon after antibiotic treatment has stopped, or re-infection from an environmental source.

Even though consensus regarding factors associated with CDI recurrence is not universal learning algorithms have been developed to predict CDI recurrence with good sensitivity [128].

Ultimately distinction between recurrence and reinfection can only be achieved if the strain of *C. difficile* is ‘typed’ using molectular epidemiology [129].

## Wider consequences of CDI

Patients who develop CDI have increased hospital length-of-stay, higher medical care costs, more hospital re-admissions, and higher mortality [130–132].

These consequences are also found for surgical patients with CDI.

In the Zerey et al. analysis [8] epidemiologic data suggested that the infection was most prevalent after emergency operations and among patients having intestinal tract resections. Infection with *C. difficile* was an independent predictor of increased length of stay, which increased by 16.0 days (95 % CI 15.6, 16.4 days; p < 0.0001) in the presence of infection. Total charges increased by $77,483 (95 % CI $75,174, $79,793; p < 0.0001), and there was a 3.4-fold increase in the mortality rate (95 % CI 3.02, 3.77; p < 0.0001) compared with patients who did not acquire *C. difficile*.

In the Abdelsattar et al. study [11] three procedure groups had higher odds of postoperative CDI: lower-extremity amputations (adjusted odds ratio [aOR], 3.5; *P* = .03), gastric or esophageal operations (aOR, 2.1; *P* = .04), and bowel resection or repair (aOR, 2; *P* = .04). Postoperative CDI was independently associated with increased length of stay (mean, 13.7 days vs 4.5 days), emergency department presentations (18.9 vs 9.1 %) and readmissions (38.9 vs 7.2 %, all P < .001).

Data from Nationwide Inpatient Sample database in patients who underwent vascular surgery [79], showed that in 2011 patients who had experienced CDI had median length of stay 15 days (IQR 9, 25 days) compared with 8.3 days for matched patients without CDI, in-hospital mortality 9.1 % (compared to 5.0 %), and $13,471 extra cost per hospitalization. The estimated cost associated with CDI in vascular surgery in the United States was about $98 million in 2011. Data from the National Inpatient Sample examined just patients with lumbar surgery and found CDI increased length of stay by 8 days, hospital costs by 2-fold and increased inpatient mortality by 36-fold [133].

Higher mortality was also observed for liver transplant recipients (from 2000 to 2010) at Detroit hospital [134].

The ACS-NSQIP database from 2005 to 2010 was used by Lee et al. to study emergently performed open colectomies for a primary diagnosis of *C difficile* colitis in US [135]. The overall mortality was 33 % (111/335). Age 80 years or older, preoperative dialysis dependence, chronic obstructive pulmonary disease), and wound class III were associated to patients mortality. Thrombocytopenia (platelet count < 150 × 10^3^/mm^3^), coagulopathy (International Normalized Ratio > 2.0), and renal insufficiency (blood urea nitrogen > 40 mg/dL) were associated with a higher mortality as well.

Recently a study was performed to quantify additional hospital stay attributable to CDI in four European countries, by analyzing nationwide hospital-episode data [5]. Patients in England had the longest additional hospital stay attributable to CDI at 16.09 days, followed by Germany at 15.47 days, Spain at 13.56 days, and The Netherlands at 12.58 days, derived using regression analysis. Propensity score matching indicated a higher attributable length of stay of 32.42 days in England, 15.31 days in Spain, and 18.64 days in The Netherlands. Outputs from this study consistently demonstrate that in European countries, for patients whose hospitalization is complicated by CDI, the infection causes a statistically significant increase in hospital length of stay.

## Recommendations for the management of CDI

### Diagnosis

**1) Stool testing should only be performed on diarrhea stools from at-risk patients with clinically significant diarrhea (Recommendation 1 C).**

**2) For patients with ileus who may be unable to produce stool specimens, polymerase chain reaction testing of perirectal swabs may be an accurate and efficient method to detect toxigenic*C. difficile*in patients with symptoms of CDI (Recommendation 2B).**

Prompt and precise diagnosis is important for the effective management of CDI.

Early identification of CDI allows early treatment and can potentially improve outcomes. Rapid isolation of infected patients is important in controlling the transmission of *C. difficile* [136]*.*

The diagnosis of CDI is based on the presence of a clinical picture compatible with CDI and microbiological evidence of free toxin and/or the demonstration of toxigenic *C. difficile* in a diarrhea stool sample [136]. Clinical features include: diarrhea (defined as by passage of 3 or more unformed stools in 24 h), abdominal pain and cramps, abdominal distension, ileus (signs of severely disturbed bowel function) and toxic megacolon.

Since *C. difficile* can colonize the intestinal tract of healthy individuals, diagnostic testing for CDI should be performed only on diarrhea stools from symptomatic patients. Testing of formed stool can result in false positive tests, which may result in unnecessary antibiotic therapy.

One limitation of the reliance on stool specimens are the patients with suspected severe CDI complicated by ileus as these patients may be unable to produce specimens for testing. For these patients testing of perirectal swabs may be an accurate and efficient method to detect toxigenic *C. difficile*. In 2012 Kundrapu et al. [137] described the results of a prospective study of 139 patients being tested for *Clostridium difficile* infection by polymerase chain reaction. The sensitivity, specificity, positive predictive value, and negative predictive value of testing perirectal swabs were 95.7, 100, 100, and 99.1 %, respectively. The authors concluded that for selected patients, perirectal swabs provided an acceptable alternative to stool specimen analysis. Clinical context such as a history of recent antibiotic administration and/or residence in hospital are useful in selecting patients for testing. Other signs such as fever, abdominal pain, leukocytosis, in combination with other laboratory tests (e.g. creatinine and serum lactate) are useful for defining severity of infection.

**3) Nucleic acid amplification tests (NAAT) such as polymerase chain reaction (PCR) for*C. difficile*toxin genes appear to be sensitive and specific and may be used as a standard diagnostic test for CDI. NAAT as single-step algorithm can increase detection of asymptomatic colonization therefore it should only be performed in patients with clinical suspicion for CDI (Recommendation 1 B).**

**4) Glutamate dehydrogenase (GDH) screening tests for*C. difficile*are sensitive but do not differentiate between toxigenic and non-toxigenic strains. They may be used in association with toxin A and B EIA testing. Algorithms involving screening with an EIA for GDH followed by a toxin assay may be used (Recommendation 1 B).**

**5) Enzyme immunoassay (EIA) for toxin A/B is fast and inexpensive and has high specificity but it is not recommended alone due to its relatively low sensitivity. (Recommendation 1 B).**

**6)*Clostridium difficile*culture is relatively slow but sensitive. It is rarely performed today as a routine diagnostic test.*C. difficile*culture is recommended for subsequent epidemiological typing and characterization of strains (Recommendation 1 C).**

**7) Repeat testing within 7 days should not be performed on patients who previously tested negative unless the clinical picture has changed significantly (Recommendation 1 C).**

The best standard laboratory test for diagnosis of CDI has not been clearly established [138]. In the past, toxigenic culture (TC) was accepted by many microbiologists as the method of choice for diagnosis of CDI. The procedure includes stool culture for *C. difficile* on a selective differential medium (cycloserine, cefoxitin, fructose agar or CCFA) and an assay to test the colonies for the ability to produce toxins. Despite the fact that TC is considered a gold standard method, there are significant issues including slow turnaround time and it’s inability to detect the presence of toxins in stool. This may also lead to false positive results given up to 7 % of asymptomatic hospitalized patients may be colonized with toxigenic *C. difficile* [139].

However, TC can still be used as a confirmatory test in symptomatic patients with toxin positive/GDH assay(s)-negative stool samples. *C. difficile* culture is also necessary for subsequent epidemiological typing and characterization of strains.

The EIA for toxin A/B has been adopted by most clinical laboratories because it is fast, convenient and inexpensive [140]. However, studies have shown that sensitivity can be low. Toxin A + B EIA tests have a described sensitivity of 32–98 % and a specificity of 84–100 % [141].

Glutamate dehydrogenase (GDH) is an enzyme produced by *C. difficile* in relatively large amounts compared with toxins A and B [142, 143]. A positive GDH assay only documents the presence of *C. difficile* but it does not discriminate between toxigenic and non-toxigenic strains (about 20 % of the *C. difficile* population). Therefore, a second test for toxin production is necessary for confirmation. GDH screening tests for *C. difficile* used in association to toxin A + B EIA testing gives an accurate test result quickly [140, 141] even if the sensitivity of such strategy is lower than nucleic acid amplification tests (NAATs).

NAATs such as PCR for CD toxin genes have a high sensitivity and specificity, but not all laboratories routinely perform this assay [143]. A current topic of debate is whether a stool sample that was positive by a molecular assay needs to be tested with a confirmatory toxin assay [144] given it can also identify toxigenic *C difficile* in asymptomatic patients. This underscores the importance of only testing patients with symptoms. There is no evidence suggesting that surgical patients should be diagnosed any differently than general medical patients.

**8) Immunocompromised patients (including patients in chemotherapy, chronic corticosteroid therapy, or immunosuppressive agents, and post-transplant patients) should be always tested for CDI if they have a diarrheal illness (Recommendation 1 C).**

It has already been highlighted that immunocompromised patients including those on glucocorticoids, or chemotherapy and post-transplant patients are at increased risk for CDI.

**9) CT imaging is suggested for suspected severe-complicated*C. difficile*colitis, however its sensitivity is not satisfactory for screening purposes (Recommendation 2 B).**

CT has been studied as an imaging modality for diagnosing *C. difficile* colitis [145–148]. Typical CT findings of CDC include colonic wall thickening, dilation, pericolonic stranding, “accordion sign” (high-attenuation oral contrast in the colonic lumen alternating with low-attenuation inflamed mucosa), “double-halo sign, target sign” (intravenous contrast displaying varying degrees of attenuation caused by submucosal inflammation and hyperemia), and ascites [149]. However, the most common finding, colonic wall thickening is non-specific and can be found in other forms of colitis, although it may be more pronounced in that caused by *C. difficile*.

In the Kirkpatrick et al. study [150], CT diagnosis of CDC was made with a sensitivity of 52 %, a specificity of 93 %, and positive and negative predictive valued 88 %, and 67 % respectively. Sensitivity would have been increased to 70 % with no change in specificity if a colon wall thickness of greater than 4 mm had been used, in conjunction with the presence of colon wall nodularity, accordion sign, peri-colonic stranding, or otherwise unexplained ascites.

**10) Ultrasound may be useful in critically ill patients suspected to have pseudomembranous colitis who cannot be transported for CT scan (Recommendation 2 C).**

Point-of-care ultrasound may be useful in diagnosing and managing critically ill patients who cannot be moved to the radiology department [151].

Ultrasound findings of pseudomembranous colitis in severe cases include a thickened colonic wall with heterogeneous echogenity and narrowing of the colonic lumen [152]. Pseudomembranes can also be visualised as hyperechoic lines covering the mucosa [152–155].

In the early stages of pseudomembranous colitis, the texture of the colonic wall is preserved. The hypoechoic edematous mucosa and muscularis propria may be thickened with the echogenic submucosa sandwiched between them. The presence of submucosal gaps may indicate extension of tissue damage into deeper structures. Intraperitoneal free fluid is seen in more than 70 % of cases [153–155].

**11) Flexible sigmoidoscopy may be helpful for the diagnosis of*C. difficile*colitis (CDC) when there is a high level of clinical suspicion for*C. difficile*despite repeated negative laboratory assays (Recommendation 2 B).**

Endoscopy should be used sparingly to confirm the diagnosis of *C. difficile* colitis since the diagnosis can be usually made by laboratory tests, clinical findings and imaging. Moreover colonoscopy may be hazardous in the setting of fulminant colitis where there may be increased risk of perforation [156].

A study by Johal et al. [157] described the use of flexible sigmoidoscopy as a tool for the diagnosis of *C. difficile* colitis when stool assays were negative. Of 136 patients with *C. difficile* associated diarrhea (CDAD) 56 patients had pseudomembranous colitis at sigmoidoscopy. The stool *C. difficile* cytotoxin test was negative in 29 (52 %) but toxigenic *C. difficile* was isolated from all of nine stool samples cultured. Of patients with pseudomembranous colitis, 30.4 % relapsed over the subsequent 57.7 days. The authors concluded that sigmoidoscopy should be considered in all hospitalised patients with diarrhea in whom the stool tests for *C. difficile* cytotoxin and enteric pathogens are negative.

Emergency colonoscopy or sigmoidoscopy may also reveal pseudomembranous colitis in patients too ill to wait for laboratory results.

## Antimicrobial therapy

**12) Unnecessary antimicrobial agent(s) and proton pump inhibitors should be discontinued if CDI is suspected (Recommendation 1 C).**

**13) Empirical therapy for CDI should be avoided unless there is a strong suspicion for CDI. If a patient has a strong suspicion for CDI, empirical therapy for CDI should be considered while awaiting test results (Recommendation 1 B).**

In cases of suspected severe CDI, antimicrobial agent(s) should be discontinued, if possible [158].

A meta-analysis addressing factors associated with prolonged symptoms and severe disease due to *Clostridium difficile* showed that continued use of antimicrobials for infections other than CDI is significantly associated with an increased risk of CDI recurrence [159].

When antimicrobial therapy is indicated for symptomatic cases with a positive *C. difficile* toxin result, options include metronidazole, oral or intraluminal vancomycin and fidaxomicin [160–166].

**14) Metronidazole is recommended for the treatment of mild-moderate disease (Recommendation 1 A).**

Given at a dose of 500 mg orally 3 times a day for 10 days, metronidazole has been shown to be an inexpensive and effective treatment of non-severe CDI [167]. Metronidazole can also be administrated intravenously with or without intraluminal vancomycin in patients unable to take oral medication e.g. those with post-surgical ileus.

A Cochrane analysis published in 2011 [167] reviewed 15 studies on the antibiotic treatment for CDI in adults. In three randomized controlled trials comparing symptomatic cure between metronidazole and vancomycin, no statistically significant difference was found [167]. Symptomatic cure was achieved in 79 % of patients who received vancomycin compared with 71 % of patients who received metronidazole (three studies; 335 patients; RR 0.91; 95 % CI 0.81–1.03, p 0.14).

**15) Oral vancomycin is recommended for treatment of patients with severe disease, or for patients with mild-moderate disease who do not respond to metronidazole. (Recommendation 1 A).**

Vancomycin orally 125 mg four times daily for 10 days is considered superior to metronidazole in severe *C. difficile* disease [168–170]. This may reflect the superior pharmacokinetic properties of vancomycin which is concentrated in the gut lumen. Doses of up to 500 mg have been used in some patients with severe CDI [7] although there is little evidence for this in the literature.

**16) In patients in whom oral antibiotics cannot reach the colon, vancomycin may be administered by enema and metronidazole can be given intravenously (Recommendation 1 B).**

Intravenous vancomycin has no effect on CDI since the antibiotic is not excreted into the colon. Vancomycin enema may be an effective therapy for patients who cannot tolerate the oral preparation or patients with ileus who have delayed passage of oral antibiotics from the stomach to the colon. Trans-stoma vancomycin may also be effective in surgical patients with Hartmann resection, ileostomy, or colon diversion. A single-hospital, retrospective chart review on 47 consecutive patients with *C. difficile* colitis treated with intra-colonic vancomycin (ICV) was published by Kim PK et al. in 2013 [171]. Thirty-three of 47 patients (70 %) with severe *C. difficile* colitis responded to adjunct ICV with complete resolution without surgery. Multivariable analysis suggested that failures to intra-colonic vancomycin enemas occurred in patients who were older and frail with albumin < 2.5 g/dl and early surgery should be considered for those patients. Early surgery should also be offered to those patients who are failing maximal medical therapy that include ICV enemas.

**17) Fidaxomicin may be used to treat CDI, especially in the patients at higher risk for recurrence (e.g. elderly patients with severe underlying disease or those requiring receiving concomitant antibiotics) (Recommendation 1 A).**

Fidaxomicin orally 200 mg twice daily for 10 days may be an alternative to vancomycin in some patients with CDI [172, 173].

Fidaxomicin was non-inferior to vancomycin for initial cure of CDI in two prospective trials [164, 165]. In a first double-blind, randomized, non-inferiority trial [164] 629 adults with acute symptoms of *C. difficile* infection and a positive result on a stool toxin test were enrolled and randomly assigned to receive fidaxomicin (200 mg twice daily) or vancomycin (125 mg four times daily) orally for 10 days. The rates of clinical cure with fidaxomicin were non-inferior to those with vancomycin in both the modified intention-to-treat analysis (88.2 % with fidaxomicin and 85.8 % with vancomycin) and the per-protocol analysis (92.1 % and 89.8 %, respectively). Significantly fewer patients in the fidaxomicin group than in the vancomycin group had a recurrence of the infection, in both the modified intention-to-treat analysis and the per-protocol analysis. In a second multi-centre, double-blind, randomized, non-inferiority trial [165] 535 patients, 16 years or older with acute, toxin-positive C difficile infection were randomly allocated (1:1) to receive oral fidaxomicin (200 mg every 12 h) or oral vancomycin (125 mg every 6 h) for 10 days. Non-inferiority was shown for both the modified intention-to-treat analysis (15.4 % vs. 25.3 %, *P* = 0.005) and the per-protocol analysis (13.3 % vs. 24.0 %, *P* = 0.004). Patients receiving concomitant antibiotics for other infections had a higher cure rate with fidaxomicin (46 [90 · 2 %] of 51) than with vancomycin (33 [73 · 3 %] of 45; *p* = 0 · 031). Fidaxomicin may be useful for treating patients who are considered at high risk for recurrence (elderly patients with multiple comorbidities who are receiving concomitant antibiotics). However, it is important to note that there are no data available on the efficacy of Fidaxomicin in severe life-threatening disease.

The use of other antibiotics such as tigecycline [174, 175] fusidic acid, teicoplanin, rifamixin [167] and nitazoxanide [176], has been described in the literature, but they are not currently recommended for general use.

## Surgical management

Patients with fulminant colitis (FC) who progress to systemic toxicity require surgical intervention.

To determine clinical predictors for the development of fulminant colitis in patients with CDI a 10-year retrospective review of FC patients who underwent colectomy was performed and compared with randomly selected age- and sex-matched non-fulminant CDI patients at a single institution study by Girotra in 2012 [177]. Predictive clinical and laboratory features included: old age (>70 years), prior CDI, profound leukocytosis (>18,000/mm^3^), hemodynamic instability, use of anti-peristaltic medications, and a clinical triad of increasing abdominal pain, distention and diarrhea.

**18) Patients with severe CDI who progress to systemic toxicity should undergo early surgical consultation and evaluated for potential surgical intervention (Recommendation 1 C).**

Patients with severe CDI who progress to systemic toxicity are likely to have serious comorbidities. Delaying surgery in this group leads to increased likelihood of adverse outcomes [178], although some reports show that a short period of medical optimization can improve outcomes before colectomy [179].

There are no reliable clinical and/or laboratory findings that can predict those patients who will respond to medical therapy and those who will subsequently need surgery [180].

Data comparing mortality rates between surgical and medical treatment for fulminant *C. difficile* colitis were published in a recent systematic review by Stewart et al. [181]. Five hundred and ten patients with FC were identified in six studies. Emergency colectomy for patients with FC provided a survival advantage compared with continuing antibiotics. When all six studies numbering 510 patients were analysed, the pooled adjusted odds ratio of mortality comparing surgery with medical therapy, and weighted by the contribution of each study, was 0.70 (0.49–0.99) leading the authors to conclude that emergency colectomy has a therapeutic role in treating complicated *C. difficile* colitis.

Patients presenting with organ failure (acute renal failure, mental status changes, or cardiopulmonary compromise) also need prompt intervention.

The timing of surgical intervention is the key for survival of patients with FC [182–185].

Seder et al. [186] described 6,841 patients with CDI and showed a decreased mortality associated with surgery performed before the need for vasopressor requirement, especially in the patients <65 years old. Hall et al. [184] reviewed 3,237 consecutive cases of CDI and showed an increased mortality rate when surgical exploration was performed after intubation or the development of respiratory failure and the use of vasopressors.

Recently a risk scoring system (RSS) for daily clinical practice was designed by van der Wilden et al. [187]. Age greater than 70 years was assigned 2 points, white blood cell count equal to or greater than 20,000 x 10^9^/L or equal to or less than 2,000 x 10^9^/L was assigned 1 point, cardiorespiratory failure was assigned 7 points, and diffuse abdominal tenderness on physical examination was assigned 6 points. A value of 6 points was determined to be the threshold for reliably dividing low-risk (<6) from high-risk (≥6) patients. Only patients with cardiorespiratory failure or diffuse abdominal tenderness were high risk.

Ferrada et al. [188] reviewed the existing literature on the treatment of CDI and published practice management guidelines (PMG) for the Eastern Association for the Surgery of Trauma (EAST). The authors strongly recommended, that adult patients with CDI undergo early surgery before developing shock and the need for vasopressors. Although timing remains controversial Ferrada et al. found that it was between 3 days and 5 days after diagnosis in patients who are worsening or not clinically improving [188].

Many factors have been described as predictors of mortality in patients who undergo emergency intervention.

Sailhamer et al. [189] reviewed the records of 4796 inpatients diagnosed with *C difficile* colitis. In 199 patients (4.1 %) with fulminant *C difficile* colitis the in-hospital mortality rate was 34.7 %. Independent predictors of mortality included age 70 years or older, severe leukocytosis or leukopenia (white blood cell count, >or = 35 000 × 10^9^/L or <4000 × 10^9^/L) or bandemia (neutrophil bands, >or = 10 %), and cardiorespiratory failure (intubation or vasopressors). Survival rates were higher in patients who were cared for by surgical vs nonsurgical departments.

The ACS-NSQIP database from 2005 to 2010 was used by Lee et al. to study emergency open colectomies performed for *C difficile* colitis in the USA [190]. The overall mortality was 33 % (111/335). Age 80 years or older, preoperative dialysis dependence, chronic obstructive pulmonary disease, and wound class III were associated high patient mortality. Thrombocytopenia (platelet count < 150 × 10^3^/mm^3^), coagulopathy (International Normalized Ratio > 2.0), and renal insufficiency (blood urea nitrogen > 40 mg/dL) were also associated with a higher mortality.

A systematic review and meta-analysis of outcomes following emergency surgery for *C. difficile* colitis was published by Banghu et al. [191]. Thirty-one studies were included, which presented data for 1433 patients undergoing emergency surgery for *C. difficile* colitis. It concluded that the strongest predictors for postoperative death were those relating to preoperative physiological status: preoperative intubation, acute renal failure, multiple organ failure and shock requiring vasopressors.

**19) Resection of the entire colon should be considered to treat patients with fulminant colitis (FC) (Recommendation 1 B).**

**20) Diverting loop ileostomy with colonic lavage may be a useful alternative to resection of entire colon (Recommendation 2 C).**

**21) Patients with FC should be treated with high dose oral or by enema vancomycin (500 mg, 6 hourly) in combination with intravenous metronidazole (500 mg, 8 hourly). (Recommendation 1 C).**

In the Bhangu et al. meta-analysis [191] the most commonly performed operation for treatment of FC was total colectomy with end ileostomy (89 %, 1247/1401). When total colectomy with end ileostomy was not performed, reoperation to resect further bowel was needed in 15.9 % (20/ 126). In the recent meta-analysis by Ferrada et al. [188], 17 studies comparing colectomy versus other procedures or no surgery as treatment for CDI were analyzed. The authors recommended that total colectomy (vs. partial colectomy or other surgery) is the procedure of choice for patients with *C. difficile* colitis.

To evaluate the role of emergency colectomy in patients with FC, and to identify subgroups of patients that may benefit Lemontagne et al. [192] published a retrospective observational cohort study of 165 cases of FC that required ICU admission or prolongation of ICU stay in 2 tertiary care hospitals of Quebec, Canada. Eighty-seven (53 %) cases died within 30 days of ICU admission, of which almost half (38 of 87, 44 %) died within 48 h of ICU admission. The independent predictors of 30-day mortality were leukocytosis > or = 50 × 10(9)/L, lactate > or = 5 mmol/L, age > or = 75 years, immunosuppression and shock requiring vasopressors. Patients who underwent an emergency colectomy were less likely to die than those treated medically. Colectomy was more beneficial in patients aged 65 years or more, in immunocompetent patients and in patients with a leukocytosis > or = 20 × 10^9^/L or lactate between 2.2 and 4.9 mmol/L.

Diverting loop ileostomy with antegrade colonic lavage may be a colon preserving alternative to total colectomy [193, 194]. To evaluate whether a minimally invasive, colon-preserving approach may be an alternative to sub-total colectomy in the treatment of FC, a historical control group study was performed at the University of Pittsburgh Medical Center or and the Veterans’ Administration Healthcare System, Pittsburgh between June 2009 and January 2011 [193]. All patients with FC were managed by a loop ileostomy, intraoperative colonic lavage with warmed polyethylene glycol 3350/electrolyte solution via the ileostomy and postoperative antegrade instillation of vancomycin flushes via the ileostomy. Forty-two patients were treated during this time period. There was no significant difference in age, sex, pharmacologic immunosuppression, and Acute Physiology and Chronic Health Evaluation-II scores between the studied cohort and historical controls. The operation was accomplished laparoscopically in 35 patients (83 %). This treatment strategy resulted in reduced mortality compared to their historical population. Preservation of the colon was achieved in 39 of 42 patients (93 %). Of note, in this study vancomycin antegrade enemas were continued via the ileostomy every 6 h for 10 days after ileostomy formation and this likely augmented the effect of the defuctioning surgery.

## Supportive care

**22) Supportive measures, including intravenous fluid resuscitation and electrolyte replacement, should be provided to all patients with severe*C. difficile*infection (Recommendation 1 C).**

Diarrhea results in significant volume depletion and electrolyte abnormalities, and fluid and electrolyte imbalance should be promptly corrected [119, 120].

**23) Early detection of shock and aggressive management of underlying organ dysfunction are essential for optimum outcomes in patients with fulminant colitis (Recommendation 1 C).**

Early detection and prompt aggressive treatment of the underlying organ dysfunction is an essential component of improving outcome of critical ill patients [120].

Severe CDI may present with a fulminant course and may be associated with great morbidity and high mortality. Physiologic support including close invasive monitoring in an intensive care unit setting and aggressive resuscitation are often necessary in fulminant colitis.

## Recurrent *C. difficile* infection (RCDI)

Recurrence is diagnosed when CDI recurs <8 weeks after the onset of a previous episode, provided the symptoms from the previous episode resolved after completion of initial treatment and other causes have been excluded. Symptomatic recurrent *C difficile* infection (RCDI) occurs in approximately 20 % of patients and is challenging to treat [195]. Patients with recurrence of CDI should therefore be treated by clinicians who have experience in treating the infection.

**24) Agents that may be used to treat the first recurrence of CDI include metronidazole, for non-severe RCDI, and vancomycin for severe RCDI. (Recommendation 1 B).**

**25) Fidaxomicin may be used as an alternative agent (Recommendation 1 B).**

A systematic review on the treatment of RCDI was recently published [196]. Metronidazole and vancomycin have a good evidence base for use in RCDI but heterogeneity in treatment duration and treatment doses between the studies precluded robust conclusions. Fidaxomicin may also have a role in the treatment of first recurrence. Fidaxomicine was superior to vancomycin in terms of recurrences, with significantly less recurrence at 28 days. This was confirmed in some subgroup analysis [197].

**26) In subsequent recurrence of CDI (2nd or later) oral vancomycin or fidaxomicin is recommended (Recommendation 1 B).**

Vancomycin and fidaxomicin are equally effective in resolving CDI symptoms but fidaxomicin has been shown to be associated with a lower likelihood of CDI recurrence after a first recurrence [164, 165, 197]. However, there are no prospective randomized controlled trials investigating the efficacy of fidaxomicin in patients with multiple recurrences of CDI. Vancomycin is often administered using a prolonged tapered and/or pulsed regimen which may be more effective than a standard 10 to 14 day course although no RCTs have been reported [198].

## Probiotics

**27) Probiotics may be considered as an adjunctive treatment to antibiotics for immunocompetent patients with RCDI (Recommendation 2 B).**

Little evidence exists to support the use of probiotics in the first episode of CDI [116]. Two randomized controlled trials showed some effectiveness for *Saccharomyces boulardii* CNCM I-745 in recurrent CDI. The first demonstrated a lower relapse rate compared with a placebo control group (35 vs 65 % in the placebo group) [199] and the second found that the combination of *S. boulardii* (1 g/d) with high dose vancomycin (2 g/d) was more effective than high dose vancomycin and placebo (17 vs 50 % recurrence rate) [200]. Other studies with Lactobacillus strains (*L. rhamnosus* GG or *L. plantarum* 299v) were stopped prematurely due to enrollment problems [201]. Probiotics should not be administered to patients at risk of bacteraemia or fungaemia [116].

There is limited evidence to support the use of probiotics for the primary prevention of CDI from developing. A meta-analysis of 11 studies was in published 2012 [202]. Two studies showed significantly lower rates of CDI among the probiotic recipients. A meta-analysis of three studies that used the probiotic combination *Lactobacillus acidophilus CL1285* and *Lactobacillus casei LBC80R* and a combined analysis of those studies with four studies that used *Saccharomyces boulardii*, showed lower CDI rates in recipients of probiotics compared with recipients of placebo (risk ratio = 0.39; 95 % confidence interval 0.19–0.79. However, given the potential risk of bloodstream infection with these organisms further studies are warrented before their use can be recommended routinely.

## Faecal microbiota transplantation

**28) Intestinal or faecal microbiota transplantation (IMT or FMT) may be an effective option for the treatment of RCDI (Recommendation 1 B).**

Intestinal or faecal microbiota transplantation (IMT or FMT) has been considered as an alternative therapy to treat RCDI [203–208]. It involves infusing intestinal microorganisms (in a suspension of healthy donor stool) into the intestine of patients to restore the intestinal microbiota.

The rationale of FMT is that disruption of the normal balance of colonic flora allows *C. difficile* strains to grow and produce CDI. By reintroducing normal flora via donor faeces, the imbalance may be corrected, and normal bowel function re-established [203].

FMT has not been widely adopted as a therapeutic tool probably due to concerns regarding safety and acceptability [204].

A systematic literature review of IMT treatment for RCDI and pseudomembranous colitis was published in 2011 by Gough et al. [205]. In 317 patients treated across 27 case series and reports, IMT was highly effective, showing disease resolution in 92 % of cases. In those studies, 35 % of patients received IMT via enema, with a response rate of 95; 23 % patients received IMT via naso-jejunal tube by gastroscope, with a response rate of 76; and 19 % via colonoscopy, with a response rate of 89 %. Effectiveness varied by route of instillation, relationship to stool donor, volume of IMT given, and treatment before infusion.

Recently a systematic review was published by Cammarota et al. [206]. Twenty full-text case series, 15 case reports, and 1 randomized controlled study were included for the final analysis. Almost all patients treated with donors’ fecal infusion experienced recurrent episodes of CD-associated diarrhea despite standard antibiotic treatment. Of a total of 536 patients treated, 467 (87 %) experienced resolution of diarrhea. Diarrhea resolution rates varied according to the site of infusion: 81 % in the stomach; 86 % in the duodenum/jejunum; 93 % in the cecum/ascending colon; and 84 % in the distal colon. No severe adverse events were reported with the procedure.

In a recently published randomized clinical trial by van Nood et al. [208] patients with RCDI were randomised to three groups; 1) vancomycin regime only; 2) vancomycin with duodenal infused FMT and 3) vancomycin and bowel lavage. In the FMT treated group an 81% reduction in diarrhoea was observed. The FMT group were observed to have normalization of their intestinal bacterial composition which was similar to that of the donor. Although, this trial has shown exciting results, these need to be interpreted with caution as the trial included only small number of patients, was not blinded, and was aborted early due to profound differences in the groups. It has also been criticised for potentially having several potential biases.

FMT may be administered via enemas or as a slurry given via a nasogastric tube. In the fall of 2014, Youngster et al. [209] reported their experience on utilizing frozen FMT capsules in 20 patients who had RCDI. Fourteen patients (70 %) had resolution of diarrhea after the first treatment, and an additional 4 patients responded after a second treatment, for a clinical resolution rate of 90 %.

**29) FMT may be effective in immunocompromised patients and patients who have had solid organ transplants (Recommendation 2 B).**

Patients who are immunocompromised are at increased risk of CDI. During the last two years the first data on FMT in immunocompromised patients began to appear in the medical literature [210].

A multicenter retrospective series on the use of FMT in immunocompromised (IC) patients with CDI that was recurrent, refractory, or severe was published in 2014 [211]. Reasons for IC included: HIV/AIDS (3), solid organ transplant (19), oncologic condition (7), immunosuppressive therapy for inflammatory bowel disease (IBD; 36), and other medical conditions/medications (15).

This series demonstrated the effective use of FMT for CDI in IC patients with few serious adverse events or related adverse events.

## Intravenous immunoglobulin (IVIG)

**30) IVIG should only be used as adjunct therapy in patients with multiple recurrent or fulminant CDI until results from large, randomized controlled trials are available (Recommendation 2 C).**

IVIG treatment has been proposed based on the evidence that the level of immune response to *C. difficile* colonization is a major determinant of magnitude and duration of clinical manifestations. Passive immunization with IVIG has been reported to be successful in several small series. A review by Abourgergi [212] of fifteen small, mostly retrospective and non-randomized studies documented success with IVIG in the treatment of protracted, recurrent, or severe CDI. The authors concluded IVIG should only be used as adjunct therapy until results from large, randomized controlled trials are available.

## Monoclonal antibodies

**31) Infusion with monoclonal antibodies may be of use to prevent recurrences of CDI, particularly in patients with CDI due to the 027 epidemic strain (Recommendation 2 C).**

In a phase II clinical trial [213], the use of monoclonal antibodies to toxins A and B as an adjunct to antibiotics was shown to decrease recurrence rates in patients with CDI compared with placebo (7 vs. 25 % respectively; 95 % confidence interval, 7 to 29; P < 0.001). The recurrence rates among patients with the epidemic BI/NAP1/027 strain were 8 % for the antibody group compared with 32 % for placebo (*P* = 0.06); among patients with more than one previous episode of CDI*,* recurrence rates were 7 and 38 %, respectively (*P* = 0.006). The authors concluded that the addition of monoclonal antibodies against *C. difficile* toxins to antibiotic agents significantly reduced the recurrence of *C. difficile* infection. The findings of this study require confirmation before firm recommendations can be made.

## Enteral nutrition in CDI

**32) Tube feeding patients should be clinically assessed due to their risk for developing CDI (Recommendation 2 C).**

It is widely accepted that enteral nutrition (EN) maintains gut mucosal integrity which leads to decreased intestinal permeability, decreased infections, and an improved immunological status. EN during episodes of diarrhea may be well tolerated and may improve enterocyte healing and maintenance of enzyme activity [214, 215]. EN, however, has also been associated with increased risk of CDI [216]. Bliss, et al. evaluated 76 tube-fed and non tube-fed hospital patients for the development of CDI [217]. Patients were controlled for age, severity of illness and duration of hospitalization. Patients who were tube-fed were statistically more likely to develop *C difficile* associated diarrhea (20 versus 8 % *p* = 0.03). One of the reasons may be prolonged use of elemental diets. It is known that critically ill patients tolerate feeding well if the feed is given in elemental form and delivered beyond the stomach into the jejunum because it is totally absorbed within the upper small intestine [218]. Elemental diets are completely absorbed within the small intestine and therefore deprive the colonic microbiota of their source of nutrition, such as dietary fiber, fructose oligosaccharides, and resistant starch [219]. The resultant suppression of colonic fermentation may therefore lead to the disruption of the normal gut flora and the creation of a “permissive” environment for *C. difficile* colonization and subsequent infection. In feeding tube patients the conversion of elemental diet feeding to a diet containing adequate indigestible carbohydrate after the first week of critical illness may, in theory, be useful.

Recently, Puri et al. [220] reported that daily concomitant treatment with 4 g cholestyramine in patients receiving long-term intravenous ceftriaxone (2 to 4 g ceftriaxone daily, for an average of >10 weeks) was associated with CDI in only three out of 46 patients (6.5 %) compared with 23.1 % of those receiving ceftriaxone alone [221]. Cholestyramine (or colestyramine) is a hydrophilic, water insoluble, non-digestible basic anion-exchange resin which can bind luminal TcdA and TcdB.

Studies have also investigated the possible value of exogenous Phosphatidylcholene (PC) administration for reinforcement of the mucus layer [222, 223]. Mucus or “exogenous” mucus in the form of PC may have a synergistic role with secretory IgA as a barrier against *C. difficile* toxin A though additional studies are needed to demonstrate its clinical benefit before recommendations can be made [222, 223].

## Anti-motility agents

**33) The use of anti-peristaltic agents for the treatment of CDI should be discouraged. If anti-peristaltic, if used in isolation agents, are used to control persistent symptoms in patients with CDI they must always be accompanied by medical therapy (Recommendation 2 C).**

A review of the literature regarding anti-motility treatment of CDI found 55 patients with CDI who were exposed to anti-motility agents [224].

Nine patients (16 %) died, and 27 patients (49 %) had unknown outcomes. Seventeen patients (31 %) with CDI developed colonic dilation; 5 of these patients with severe CDI died. However, all patients who experienced complications or died were given anti-motility agents alone initially, without an appropriate antibiotic and 23 patients who received metronidazole or vancomycin co-administered with the anti-motility agent experienced no complications. Further study of the role of anti-motility agents in providing symptomatic relief and reducing environmental contamination with infectious stool may be warranted though, until there is clear evidence of benefit, their use in patients with CDI should be avoided [116].

## Prevention

**34) Proper antimicrobial stewardship in selecting an appropriate antibiotic and optimizing its dose and duration to cure an infection may prevent the emergence of*C. difficile*(Recommendation 1 B).**

Despite vigorous infection control measures until recently, CDI was causing an increasing problem in healthcare facilities worldwide. As CDI is thought to follow disruption to the normal bacterial flora of the colon occurring as a consequence of antibiotic use [225], it is logical that antibiotic stewardship programs may be useful in preventing CDI [226]. Good antimicrobial stewardship involves ensuring appropriate antibiotic choice and optimizing antibiotic dose and duration to cure an infection while minimizing toxicity and conditions conducive to CDI. Recently, a systematic review [227] of interventions to improve antibiotic prescribing practices for hospital inpatients suggested that reducing excessive antibiotic prescribing can prevent hospital-acquired infections and that interventions to increase effective prescribing improve clinical outcome. It would appear that cephalosporin and quinolone antibiotics may be particularly high risk, in this context [116, 228].

**35) Patients with suspected or proven CDI should be placed in contact (enteric) precautions (Recommendation 1 B).**

Prompt identification of patients with symptomatic CDI is essential so that appropriate isolation precautions can be put into effect.

This is particularly important in reducing environmental contamination as spores can survive for months in the environment [229], despite regular use of environmental cleaning agents.

Contact (enteric) precautions patients with CDI should be maintained until the resolution of diarrhea, which is demonstrated by passage of formed stool for at least 48 h. Patients with known or suspected CDI should ideally be placed in a private room [116, 230] with en-suite hand washing and toilet facilities. If a private room is not available known CDI patients may be cohort nursed in the same area [231] though the theoretical risk of transfection with different strains exists.

This is supported by a retrospective cohort of 2859 patients by Chang et al. [232]. Patients who were roommates or neighbors of a patient with CDI were at risk of nosocomial acquisition of CDI (RR, 3.94; 95 % CI, 1.27–12.24).

**36) Hand hygiene with soap and water is a cornerstone of the prevention of*C. difficile*. Hand hygiene, contact precautions and good cleaning and disinfection of the environment and patient care equipment, should be used by all health-care workers contacting any patient with known or suspected CDI (Recommendation 1 B).**

Hand hygiene with soap and water and the use of contact precautions along with good cleaning and disinfection of the environment and patient equipment, should be used by all health-care workers contacting any patient with known or suspected CDI. Hand hygiene is a cornerstone of prevention of nosocomial infections, including *C. difficile.* Alcohol-based hand sanitizers are highly effective against non–spore-forming organisms, but they may not kill *C. difficile* spores or remove *C. difficile* from the hands [233, 234].

The most effective way to remove them from hands is through hand washing with soap and water.

For environmental cleaning, hypochlorite disinfection such as sodium hypochlorite solutions are suggested for regular use in patient areas where *C. difficile* transmission is ongoing [231].

Though disposable glove use during care of a patient with CDI may be effective in preventing the transmission of *C. difficile* [230], these must be removed at the point of use and hands thoroughly decontaminated afterwards through soap and water hand washing.

## Abbreviations

CDI: *C. difficile* infection
RCDI: Recurrent *C. difficile* infection
FC: Fulminant colitis
